# Lightweight Self-Detection and Self-Calibration Strategy for MEMS Gas Sensor Arrays

**DOI:** 10.3390/s22124315

**Published:** 2022-06-07

**Authors:** Bing Liu, Yanzhen Zhou, Hongshuo Fu, Ping Fu, Lei Feng

**Affiliations:** School of Electronics and Information Engineering, Harbin Institute of Technology, Harbin 150080, China; liubing66@hit.edu.cn (B.L.); 20s005077@stu.hit.edu.cn (Y.Z.); 21s105135@stu.hit.edu.cn (H.F.); fuping@hit.edu.cn (P.F.)

**Keywords:** gas sensor arrays, Internet of Things (IoT), intelligent gas sensing system, fault detection, fault isolation and recovery, drift compensation, lightweight

## Abstract

With the development of Internet of Things (IoT) and edge computing technology, gas sensor arrays based on Micro-Electro-Mechanical System (MEMS) fabrication technique have broad application prospects in intelligent integrated systems, portable devices, and other fields. In such complex scenarios, the normal operation of a gas sensing system depends heavily on the accuracy of the sensor output. Therefore, a lightweight Self-Detection and Self-Calibration strategy for MEMS gas sensor arrays is proposed in this paper to monitor the working status of sensor arrays and correct the abnormal data in real time. Evaluations on real-world datasets indicate that the strategy has high performance of fault detection, isolation, and data recovery. Furthermore, our method has low computation complexity and low storage resource occupation. The board-level verification on CC1350 shows that the average calculation time and running power consumption of the algorithm are 0.28 ms and 9.884 mW. The proposed strategy can be deployed on most resource-limited IoT devices to improve the reliability of gas sensing systems.

## 1. Introduction

In recent years, with the rapid development of the IoT and advanced manufacturing technology, as well as the growing demand for gas detection in various fields, gas sensors have rapidly developed and have been widely used in smart terminals, environmental monitoring, disease diagnosing [1,2,3,4,5], and other fields. The accuracy and sensitivity of gas sensors have become better. In many complex application scenarios (e.g., IoT), due to the cross sensitivity and low selectivity of sensors, multiple gas sensors with different sensitive modes for the target gas are often combined into arrays for use in edge smart devices or integrated sensing systems.

Metal Oxide Semiconductor (MOS) gas sensors have been widely used in many fields [6,7] because of fast response, low cost, high sensitivity, simple circuit, etc. Traditional MOS gas sensor arrays are generally bulky, and the power consumption and cost will increase with the number of sensors. This also limits the application of MOS gas sensors in energy-efficient or portable/wearable devices, such as health and safety detection, electronic noses, and hand-held respiratory analyzers [1,8,9,10,11,12].

With the development of micro-fabrication and semiconductor technology during the past decades, the MOS gas sensor arrays based on MEMS use the wafer-level manufacturing process to make a micro-hotplate on the Si-based substrate, which greatly reduces the component volume and power consumption. Additionally, the MEMS gas sensor array is easy to be integrated with Complementary Metal Oxide Semiconductor (CMOS) circuit, which improves the compatibility between the gas sensors and Application Specific Integrated Circuit (ASIC). Therefore, MEMS gas sensor arrays have great development and application prospects in the fields of edge computing, intelligent integrated systems, and so on [13,14].

At the same time, gas sensor arrays often combine the corresponding signal processing and machine learning algorithms to form an intelligent gas sensing system for use [15,16]. In the intelligent gas sensing system, the sensor arrays respond to different target gases and generate a set of output signals, and then make classification decision or regression prediction through feature extraction and pattern recognition algorithms.

As the data acquisition device in an intelligent gas sensing system, the stability and reliability of gas sensor arrays are essential. For example, MOS gas sensors are limited by the characteristics of metal oxide materials. Irreversible chemical reactions of materials (e.g., aging and oxidation) or external interference (e.g., temperature and humidity) may cause sensor drift or failure, affect the measurement quality of the gas sensor array, and degrade the performance of subsequent algorithms, which will lead to abnormal operation of the system. In addition, considering that MEMS gas sensor arrays have the characteristics of miniaturization and integration, and in many IoT scenarios, intelligent sensor systems are often deployed in harsh environments, which means that the probability of failure for the MEMS gas sensor array is greater than that of the single sensor and traditional MOS sensor arrays. Therefore, it is necessary to adopt appropriate Self-Detection and Self-Calibration algorithms to monitor the working status of MEMS gas sensor arrays and recover the fault signal in time.

It cannot be ignored that gas sensing terminal devices in IoT or edge computing need to process the streaming response data produced by sensor arrays in real time when in use, and the computational complexity of the algorithms carried on the devices is limited by restricted computing and storage resources. Therefore, the Self-Detection and Self-Calibration algorithms are required to be lightweight, which means that they can perform real-time computation while maintaining low resource consumption and low running power consumption (mW level).

Over the years, many studies have made great efforts in realizing the monitoring of operational status of gas sensors and improving the reliability of measurement signals. For instance, a fault detection and recovery technique (FDRT) was proposed in [17] for wireless sensor network (WSN) to identify and recover the failed cluster, and ref. [18] used the structural response data measured with the redundant sensor network to detect, identify, and quantify the sensor fault. However, Refs. [17,18] are oriented to WSN, and their methods are not suitable for the operational framework of IoT and non-redundant sensor arrays. Self-validating sensor technology was adopted in [19,20] to increase the reliability of gas sensor measurements. Ref. [19] proposed a failure detection, isolation, and recovery (FDIR) strategy based on Principal Component Analysis (PCA) and Relevance Vector Machine (RVM) techniques. This strategy built a separate predicator for each sensor, which had high requirements for the computing platform. Ref. [20] used PCA and contribution plots methods to detect and isolate sensor faults. The accuracy of this method would be seriously degraded in case of multiple faults. A convolutional neural network (CNN) using the random forest (RF) classifier was used in [21] for hydrogen sensor fault diagnosis. Ref. [22] used observer-based techniques and a fault detection and isolation scheme to guarantee functional safety of sensors.

In addition, some studies have proposed adaptive correction methods to reduce the effect of sensor drift [23,24]; however, most of the models used were complex and relied on a large amount of long-term drift training data. Ref. [25] recalibrated the sensor by periodically measuring the baseline response, which was time-consuming and laborious. Estimation theory was used in [26] for online baseline drift compensation. Refs. [27,28] have proposed the signal preprocessing method that used baseline processing to offset the effect of drift. However, such methods [27,28] were generally used for data offline processing. It cannot be applied in real time, and it ignores the impact of environmental changes [26].

Unlike traditional sensor arrays, MEMS gas sensor arrays are more prone to multiple failures. Meanwhile, gas sensing terminal devices have strict requirements on speed, power consumption, and resource occupation for the deployed algorithms. However, the existing methods cannot effectively solve the above problems. Therefore, research on Self-Detection and Self-Calibration methods for MEMS gas sensor arrays is challenging and practical.

For the failure of MEMS gas sensors caused by different unstable factors (physical or chemical changes), the manifestation of the failure signal may be the same. This paper mainly focuses on five common fault manifestations of gas sensor output signals [19,20,29,30,31]. The different fault types and the corresponding fault manifestations and causes are shown in Table 1.

Considering the fault problems and resource constraints of MEMS gas sensor arrays in the process of sensing tasks of IoT terminal devices, aiming at the limitations of existing methods in architecture and application, this paper proposes a lightweight Self-Detection and Self-Calibration strategy to improve the measurement reliability and environmental adaptability of MEMS gas sensor arrays. Furthermore, an experimental system based on MEMS gas sensor arrays was built to verify the effectiveness and feasibility of the proposed method.

The specific contributions of this work are as follows:

(1) A novel and complete Self-Detection and Self-Calibration strategy for MEMS gas sensor arrays is proposed to improve the stability of the sensor arrays. The strategy includes the steps of fault detection, fault isolation, data recovery and drift compensation;
(2) A Self-Detection method combining different data preprocessing techniques is employed to realize the rapid detection of sensor array working status. This method applies a PCA-SPE model and performs appropriate preprocessing for the possible outliers and random noise in real-world dataset to improve detection accuracy;
(3) A Self-Calibration method that can cope with different fault conditions is proposed. The method adopts data reconstruction and confidence interval prediction to achieve isolation and recovery of single fault and multiple faults, and dynamic baseline differential processing is used in this method to compensate the long-term drift signal of gas sensor array in real time;
(4) The performance of the proposed methods was verified and evaluated using a variety of metrics on a real-world dataset synthesized with different sensor faults, and a comprehensive comparison with other algorithms was carried out. The comparative and board-level validation results demonstrate that our methods can operate in the gas sensing system efficiently and in a way that saves energy.

The rest of this paper is organized as follows: Section 2 introduces our Self-Detection and Self-Calibration strategy and methods in detail, including the overall process and specific algorithm principles and implementations. Then, detailed experiments and comparisons in Section 3 were used to verify the effectiveness of the proposed methods. Finally, Section 4 concludes our work.

## 2. Methodology

### 2.1. Overall Process of Self-Detection and Self-Calibration Strategy

**Figure 1:**
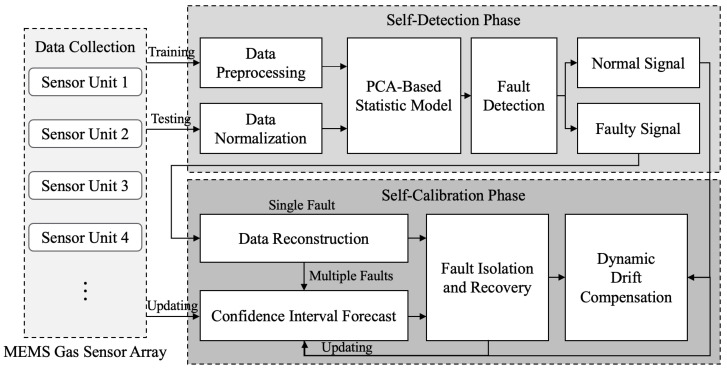
Overall process of Self-Detection and Self-Calibration strategy.

The overall process of Self-Detection and Self-Calibration is shown in Figure 1. When the input data collected by the MEMS sensor array arrives, our proposed strategy will go through two phases: Self-Detection and Self-Calibration. 

The specific process of the Self-Detection phase is as follows: Firstly, the collected data set under a normal working status is used to train the PCA-based fault detection model. To avoid the influence of the contaminated real data on the model training, different preprocessing techniques are used to improve the detection performance of model. Then, when the testing data arrives, it will undergo the same normalization as the training data. Finally, the sensor data are monitored online through the trained model to determine whether the sensor is faulty and identify input data as normal or a fault signal.

The Self-Calibration phase includes fault isolation and recovery (FIR) and dynamic drift compensation. The specific process is as follows: Firstly, the faulty signal will be assumed to be a single fault signal (because the probability of single fault is generally much higher than that of multiple faults), and then a method based on data reconstruction is used to identify a faulty sensor in the sensor array. Then, data recovery is achieved by replacing the faulty signal with the reconstructed values. However, multiple failures of MEMS sensor arrays cannot be ignored. Therefore, if the system is still judged to have faults after reconstruction, the proposed method based on confidence interval prediction will be used to solve multi-fault problem. Thus, some faulty sensors in the sensor array are identified, and the middle value of the prediction interval is used for data recovery. To ensure prediction accuracy, historical normal data and recovery data within a certain range are used to update the model.

In addition, as a long-term dynamic process, drift is a common problem in many gas sensors. In our strategy, on-line dynamic drift compensation is performed on both the normal signal and the signal after data recovery. Finally, a set of Self-Detection and Self-Calibration output signals will be provided, including original data, fault recovery data and drift compensation data, for the subsequent application algorithm to analyze.

It is worth mentioning that, considering that the possibility of single fault is relatively greater than that of multiple faults, our strategy will start with the single FIR method (data reconstruction) first when a sensor array failure is detected. If the fault is still detected after reconstruction, multiple FIR methods (confidence interval prediction) will be carried out. This operation can greatly reduce unnecessary computational burden and operating power consumption.

### 2.2. Self-Detection Methods

#### 2.2.1. Data Preprocessing of Singular Points and Random Noise

In practical application scenarios, most sensor datasets are collected in the real world. During the acquisition process, there may be unknown situations such as sudden changes in gas composition or environmental quantities (e.g., temperature and humidity), supply voltage changes, or human interference, which will lead to singular points or random noise in the original data. It can be found that if these abnormal data are not processed, problems such as missed detection or false alarm of faults will occur when the fault detection model is built.

However, most of the current fault detection research mainly focused on the design and implementation of algorithms [32,33,34]. They usually use the existing public datasets or ignore the above-mentioned problems. Some studies simply preprocess the dataset, but this usually degrades the performance of the algorithm in actual tests. Therefore, the processing of the dataset is necessary. Next, the principle and performance evaluation index of the data preprocessing methods adopted in this paper will be explained.

Singular points are “extreme values” that deviate from other normal observations of the dataset, whose values are abnormally large or small and distribution is significantly different from the overall samples [35]. In this paper, the processing of outliers is to detect the unreasonable data points of dataset and delete them through the novelty detection algorithm to avoid the impact of singular points on model training.

There are two commonly used novelty detection algorithms: statistical methods and machine learning methods. Statistical methods will build a probability distribution model based on the dataset and judge the probability that the data conforms to the model. Samples with low probability are regarded as singular points. Machine learning methods will use machine learning algorithms such as clustering or classification to judge outliers based on the distribution of data features. This paper applies Z-Score, a statistical method, to deal with outliers. In addition, another novelty detection algorithm based on machine learning (Isolation Forest) will be compared through experiments in Section 3.2, and the performance of the algorithm will be evaluated through two indicators: outlier detection rate and false positive detection rate.

Z-Score is a novelty detection method in low-dimensional data feature space [36,37]. It is derived from “3σ principle” in statistics: if the data follow a normal distribution, an outlier is defined as the value in a set of observations that deviates from the mean by more than three standard deviations of the data. In other words, outliers are usually observations near the tail of the data distribution. Therefore, singular points are usually far from the average value of data, and this distance is represented by $Z_{i}$ of the normalized dataset in Z-Score. As shown in Equation (1), where $x_{i}$ represents a data point, μ and σ represents the mean and standard deviation of the dataset. When $Z_{i}$ is greater than the set threshold $Z_{limit}$, it is determined as a singular point. The value of $Z_{limit}$ needs to be adjusted according to the distribution of the actual dataset, usually 2~3.

(1) $$Z_{i}=\frac{x_{i}-\mu }{\sigma }>Z_{limit}$$

Isolation Forest is an unsupervised novelty detection method suitable for large datasets of continuous data [38]. The method does not require labeled samples to train, but the features must be continuous. In Isolation Forest, the dataset is randomly divided recursively until all samples are isolated. Under this random segmentation strategy, the outliers usually have shorter paths.

Filtering algorithms can minimize random noise in the dataset and restore the true situation of the measured values. In this paper, the filtering algorithm is combined with the novelty detection algorithm. After removing outliers in the dataset, filtering algorithms are used to improve the quality of dataset and make it close to the real data distribution.

Commonly used filtering algorithms include Moving-Average filter, Discrete Wavelet Transform, Savitzky–Golay filter and so on [39,40,41]. The selection of filtering method depends on the characteristics of dataset and the performance of filtering. The fluctuation of gas sensor response is generally slow, and we hope that the original data features can be better preserved after filtering. Considering that a Moving-Average filter will destroy the boundary features of gas concentration changes, and Discrete Wavelet Transform needs to select or design complex wavelet basis functions, this paper adopts Savitzky–Golay filter to solve the random interference of dataset.

The Savitzky–Golay filter is a method based on local polynomials and least square fitting in time domain. The advantage of this method is that it can ensure that the shape and width of the signal remain unchanged while filtering out noise. Assuming a window of length 2*m* + 1, Savitzky–Golay performs fitting based on *k* − 1 (*k* < 2*m* + 1) degree polynomial for all measurement points in this window:

(2) $$y=a_{0}+a_{1}*x+a_{2}*x^{2}+\ldots +a_{k-1}*x^{k-1}$$

Express Equation (2) in matrix form, where the data vector is Y, the coefficient matrix is A, the independent variable matrix is X, and the residual is ε:

(3) Y=A∗X+ε

The sparse matrix $\hat{A}$ is obtained through the least square method, and the fitted data prediction value $\hat{Y}$ is shown in Equation (4). In this way, the window slides from left to right to fit all data points, thus realizing smoothing of the data.

(4) $$\hat{Y}=X*\hat{A}=X*{\left(X^{T}*X\right)}^{-1}*X^{T}*Y$$

Furthermore, to verify the filtering performance of the Savitzky–Golay filter and the ability to preserve the original data features. In this paper, the signal to noise ratio (SNR) and Pearson correlation coefficient (R) are selected as test indexes to compare the performance of Savitzky–Golay filter and Moving-Average filter. The SNR measures the size of the noise component in the filtered data, which can evaluate the quality of filtering. The Pearson correlation coefficient represents the correlation between the filtered data and the original data, which can evaluate the ability of filter to retain the data features. The calculation of SNR and R is shown in Equations (5) and (6), where $P_{s}$ is the original signal power, $P_{n}$ is the noise signal power, X and Y are the original dataset and the filtered dataset, respectively.

(5) $$\mathrm{SNR}=10lg\left(\frac{P_{s}}{P_{n}}\right)\left(\mathrm{dB}\right)$$

(6) $$R=\rho_{X,Y}=\frac{cov\left(X,\;\;Y\right)}{\sigma_{x}\sigma_{Y}}=\frac{E\left[\left(X-\mu_{X}\right)\left(Y-\mu_{Y}\right)\right]}{\sigma_{x}\sigma_{Y}}$$

#### 2.2.2. Fault Detection Based on PCA-SPE Model

Gas sensor arrays usually contain several different types of sensor elements, and the signals output by the array often related to each other. As a data-driven multivariate analysis method, PCA can express the internal relationship of multiple variables in a low-dimensional manner to obtain potential connections between variables. In addition, PCA-based fault detection methods use historical normal datasets to build statistical model, which takes up less memory and has fast computation speed. Therefore, this section studies the method based on PCA to realize the online monitoring of the working status of MEMS gas sensor array.

When the MEMS sensor array is running normally, it is assumed that there are *n* sensors in the array, each sensor has taken *m* samples at different calibration points, and the training dataset $x_{m\times n}$ is collected. This dataset contains the output signal of the sensor array at different times of the calibration point. Fault detection based on PCA is to establish the relationship model between sensors in the array according to the normalized dataset under normal working status and then judge whether the fault occurs by the change value of the statistics in the projection space.

The fault detection process based on PCA is as follows: Firstly, standardize the sample matrix $x_{m\times n}$, and obtain the data matrix $X_{m\times n}$ after processing. The PCA is then performed, as shown in Equations (7) and (8). Obtain the correlation coefficient matrix R of $X_{m\times n}$, and then perform singular value decomposition on R to obtain the eigen value matrix $\Lambda =\mathrm{diag}\left(\lambda_{i},\;i=1,2,\ldots ,n\right)$ and the eigen vector matrix $P=\left[p_{1},p_{2},\ldots ,p_{n}\right]$.

(7) $$R=\frac{X^{T}X\;}{m-1}$$

(8) $$R=P\Lambda P^{T}$$

Then, as shown in Equation (9), according to the Cumulative Percent Variance (CPV), the first *k* principal components are selected for dimensionality reduction, and the fault detection model of sensor array is built. In this paper, CPV is 0.95.

(9) $$\mathrm{CPV}=\overset{k}{\underset{i=1}{\sum }}\lambda_{i}\;/\;\overset{m}{\underset{i=1}{\sum }}\lambda_{i}\times 100\%$$

As shown in Equations (10) and (11), $X_{m\times n}$ is projected into the Principal Component Subspace (PCS) and the Residual Subspace (RS). The space composed of the first *k* linearly independent eigen vectors $\hat{P}$ is PCS, and the space composed of the last *n-k* linearly independent eigen vectors $\tilde{P}$ is RS.

(10) $$\hat{P}=\left[p_{1}\;p_{2}\ldots \;p_{k}\right]$$

(11) $$\tilde{P}=\left[p_{k+1}\;p_{k+2}\ldots \;p_{m}\right]$$

Transform the samples into PCS and RS by projection matrix *C*:

(12) $$X=\hat{X}+\tilde{X}$$

(13) $$\hat{X}=CX=\hat{P}{\hat{P}}^{T}X$$

(14) $$\tilde{X}=\left(I-C\right)X=\tilde{P}{\tilde{P}}^{T}X$$

where $\hat{X}$ and $\tilde{X}$ are the projections of X in PCS and RS, respectively.

The PCA-based fault detection model uses process statistics in PCS or RS to measure the projected changes of the samples to be tested in these two spaces. The change value of statistics indicates the deviation degree from the normal data distribution, which is used to test whether a fault is currently occurring.

Generally, Squared Prediction Error (SPE) and Hotelling’s T^2^ (T^2^) statistics were used to detect whether the system is abnormal [19,42,43]. In practical application, it is necessary to select SPE or T^2^ according to the actual situation of dataset. Squared Prediction Error is more sensitive to faults, so it is more capable of detecting faults of the same magnitude than T^2^. Considering the weak amplitude and variation of the collected gas response data, SPE statistic is selected in our work to realize the fault detection of MEMS sensor array. The experimental section later in this paper will also compare and verify the detection performance of these two statistics.

The calculation of the SPE statistic is shown in Equation (15). Squared Prediction Error measures the change degree in the projection of observed samples in RS. Normally, because the correlation between variables in RS is weak, the relative variation of data in RS is not obvious. When the sensor of array fails, the correlation between variables changes, and the established PCA model cannot adapt to the existing correlation, resulting in the increase in SPE. When SPE exceeds the control limit $\delta_{\alpha }$, the sensor array is considered to be faulty, otherwise it is in a normal working status. The detailed calculation process of $\delta_{\alpha }$ is shown in Equations (16)–(18), where $C_{\alpha }$ is the critical value of confidence level α of normal distribution.

(15) $$\mathrm{SPE}={|\left|\tilde{X}\right||}^{2}=X_{i}^{T}\left(I-C\right)X_{i}\leq \delta_{\alpha }$$

(16) $$\delta_{\alpha }=\theta_{1}\;{\left[1+\frac{C_{\alpha }h_{0}\sqrt{2\theta_{2}}}{\theta_{1}}+\frac{\theta_{2}h_{0}\left(h_{0}-1\right)}{\theta_{1}^{2}}\right]}^{\frac{1}{h_{0}}}$$

(17) $$\theta_{i}=\overset{n}{\underset{j=k+1}{\sum }}\lambda_{j}^{i},\;\;i=1,2,3$$

(18) $$h_{0}=1-\frac{2\theta_{1}\theta_{3}}{3\theta_{2}^{2}}$$

**Figure 2:**
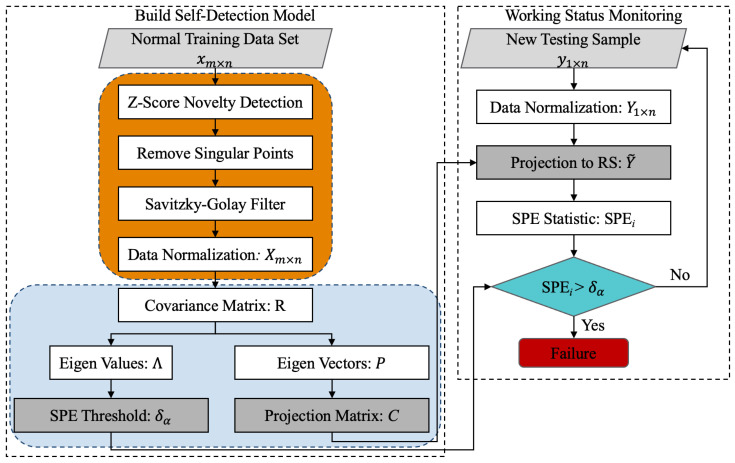
Flowchart of Self-Detection.

Figure 2 shows the flowchart of the Self-Detection method proposed in this paper. The left side is the establishment process of Self-Detection model, and the right side is the monitoring process of sensor working status using the trained model. This method uses SPE statistics as the stop criterium to control the procedure. When the MEMS sensor array fails, the correlation between variables changes, and the established PCA statistical model cannot adapt to the existing correlation, resulting in the increase in SPE*_i_*. When SPE*_i_* exceeds the control limit $\delta_{\alpha }$, it is considered that the sensor array is faulty. The Self-Detection procedure is stopped at this time and the Self-Calibration process will be carried out. The specific process of Self-Detection method is as follows:

(1) The singular points in the original normal dataset $x_{m\times n}$ are detected and removed by Z-Score algorithm, and then the noise components are removed by the Savitzky–Golay filter, and the processed data are normalized to $X_{m\times n}$;
(2) The covariance matrix R of $X_{m\times n}$ is obtained by Equation (8) and eigen values Λ and eigen vectors P can be obtained by singular value decomposition;
(3) The number of principal components *k* is obtained according to CPV, and the threshold $\delta_{\alpha }$ of SPE statistic and projection matrix *C* are calculated;
(4) When the test sample $y_{1\times n}$ is input, use the mean and variance in step (1) to normalize the testing data to obtain $Y_{1\times n}$;
(5) Project the testing sample into RS through *C*, calculate and compare SPE*_i_* and $\delta_{\alpha }$ to judge whether a fault occurs;

### 2.3. Self-Calibration Methods

When the fault of the MEMS sensor array is detected, it is necessary to isolate the fault sensor, that is, to determine the location of the fault sensor in array. Then, the fault data are recovered according to the normal data or the historical recovery data. Finally, whether it is normal or faulty sensor data, drift compensation will be carried out to complete the Self-Calibration of the sensor array.

Most of the existing research on sensor fault isolation and recovery has focused on the case of single fault [32,44]. This is because the possibility of multiple faults occurring in the sensor array is relatively small, so the problem of multiple faults is rarely considered. However, due to the characteristics of miniaturization and high integration, the probability of multiple faults of the MEMS gas sensor array is much higher than that of traditional sensor arrays. Therefore, in addition to single fault, this section will also focus on the case of multiple faults.

**Single FIR**: Contribution plots is a commonly used fault isolation method [45]. It realizes fault isolation by calculating the contribution rate of each variable to the change of fault detection statistics. However, this method has a tailing effect, which may lead to low accuracy. At the same time, most fault recovery algorithms were based on artificial neural network (ANN) [46]. This method requires many historical samples to establish and constantly update the model. However, most of the collected gas response datasets have few calibration points and weak signal change. It is difficult to meet the requirements of ANN for training samples, which will lead to low data recovery accuracy.

In addition, most of the current data recovery algorithms (ANN, RVM [19], etc.) need to build a separate prediction model for each sensor in array. In the prediction process, many historical data must be used to constantly update the model, which increases the computational burden of system. The computation and storage resources of gas sensing systems in IoT scenarios are extremely limited, which will make these models difficult to meet the requirements of real-time computing, or even unable to be deployed. Therefore, the method of data reconstruction based on SPE statistics is studied in Section 2.3.1 to realize single FIR.

**MultipleFIR**: At present, there is relatively little research in this field. The existing algorithms are like the data recovery algorithms in the case of single FIR. These methods will build a separate model (e.g., ANN, RVM) for each sensor and then isolate and recover faults according to the deviation between the predicted values of model and the original measured values.

However, these existing algorithms for multiple FIR occupy too much memory and are too computationally complex to be deployed in edge or portable gas sensing systems. Therefore, Section 2.3.2 proposes a multiple FIR method based on confidence interval prediction. The data recovery accuracy of this algorithm is high, and the parameters and computation of this algorithm are much less than that of ANN, RVM, and so on. The most important thing is that this method can realize real-time fault isolation and recovery on the resource-limited terminal devices.

**Drift Compensation**: Baseline drift [23,24,25,26,27,28] of gas sensors may be caused by slow changes in the environment, sensor aging, etc. Sensor drift causes input-output characteristics to change over time. This phenomenon affects the selectivity and sensitivity of the gas sensor, which may result in wrong response. To solve the above problems, an online compensation method for baseline drift is proposed in Section 2.3.3. It uses a dynamic matrix to update and store the baseline response according to environmental changes, and then compensates in real-time for the baseline drift by means of differential processing, thereby improving the stability of the sensor array over time.

#### 2.3.1. Single FIR Based on Data Reconstruction

The fault sample vector can be expressed as:

(19) $$X_{i}=\bar{X_{i}}+f\varepsilon$$

where $\bar{X_{i}}$ is the normal component of $X_{i}$, f is the fault amplitude component, ε is the unit matrix of fault direction, and its non-zero element $f_{i}$ represents the fault sensor *i*.

**Figure 3:**
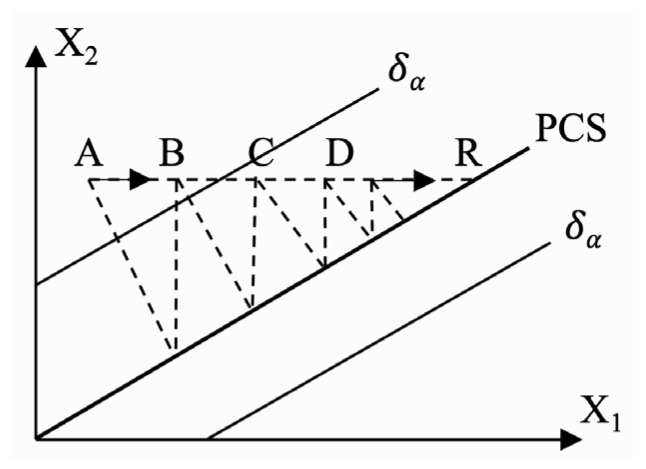
Schematic diagram of data reconstruction.

As shown in Figure 3, the idea of data reconstruction is based on Equation (13). The observation value of fault sensor is projected along the fault direction vector into PCS of the established PCA model during fault detection. According to the theory of PCA, the reconstructed value obtained by projection can be considered as the best estimation of fault recovery data. This method is simple, direct, and has few computations. However, this method applies fault measurements when calculating the projection, which may bring reconstruction error. In this section, multiple iterations are used for reconstruction to reduce the interference of faulty data, while the general reconstruction method only iterates once [32]. The formula for loop iteration is as follows:

(20) $$\hat{x_{i}}=Cx_{i}=\left[x_{1},x_{2},\ldots ,x_{m}\right]{\left[c_{1i},c_{2i},\ldots ,c_{mi}\right]}^{T}=\left[c_{-i}^{T}\;0\;c_{+i}^{T}\right]x+c_{ii}x_{i}$$

where $\left[c_{-i}^{T}\;0\;c_{+i}^{T}\right]$ represents the vector which the *i*-th column of matrix C is replaced by zero.

According to the orthogonality of matrix *P*, it can be proved that $c_{ii}$ is less than 1, then $\hat{x_{i}}$ can finally converge. That is, when $\hat{x_{i}}=x_{i}$, the formula of final iterative reconstruction can be obtained:

(21) $$\hat{x_{i}}=\frac{{\left[c_{1i},c_{2i},\ldots ,c_{\left(i-1\right)i},0,\ldots ,c_{mi}\right]}^{T}x}{1-c_{ii}}$$

Before iterative reconstruction, because the fault direction is uncertain, we will assume a set *D =* {*d*_1_, *d*_2_, *…*} containing all fault directions (where *d_i_* is obtained by the combination function *C* (*n*, *d*), and *d* is the number of fault sensors). Then, iterate continuously according to the fault direction in set *D* until the reconstructed SPE value is lower than the control limit. At this time, the recovery is considered successful, and the fault direction and corresponding recovery data are obtained. If the reconstructed SPE value is still higher than threshold after iteration of all fault direction, it is considered that a multi-fault problem has occurred, and multiple FIR will be performed. In iterative reconstruction, the T^2^ statistic can also be used for iteration. In the experimental part of this paper, the appropriate statistic will be selected by comparison.

The data reconstruction method is mainly judged by the projection change of the reconstructed data in RS. In this method, when multiple faults occur, the reconstructed SPE value is always greater than the control limit, so it cannot effectively isolate and recover multiple faults. The multiple FIR method will be discussed in the following section.

#### 2.3.2. Multiple FIR Based on Confidence Interval Forecast Using Bootstrap

The data reconstruction method has good isolation and recovery performance in single fault. However, in the face of multi-fault problem, the number of fault direction set *D* will increase exponentially with the number of sensors, and with the increase in fault data, the recovery accuracy will decline seriously. In addition, the commonly used multiple FIR methods (e.g., ANN, RVM) are computationally complex and time-consuming, making it difficult to meet the requirement of computing speed.

To solve the above problems, we propose a multiple FIR method based on confidence interval prediction using bootstrap. This method builds the model with less historical data and applies a bootstrap-based confidence interval forecasting method of time series. In case of multiple faults, the confidence interval is the prediction of fluctuation range of the current measurement. If the current measurement value exceeds the interval range, the sensor is identified as faulty, thus completing the fault isolation. Failure recovery uses the middle of the prediction interval to replace the failure measurement. After experimental verification, this method can complete the accurate isolation and recovery of multiple faults when using less historical data. Computational speed and storage occupation are also better than other methods such as ANN or RVM.

Bootstrap-based confidence interval prediction uses the bootstrap method to simulate a set of predicted values at a certain time when the distribution of prediction errors is unknown. Then, take the quantile of prediction values set as the prediction interval according to the confidence. The prediction error $e_{t}$ is defined as:

(22) $$e_{t}=y_{t}-{\hat{y}}_{t}$$

where $y_{t}$ refers to the actual measured value, and ${\hat{y}}_{t}$ is the predicted value at the current moment using historical data.

Equation (22) can be rewritten as (23), so we can use Equation (24) to simulate the next observation. Assuming that the prediction error in the next step is similar to that in the past, then $e_{t+1}$ can be replaced by sampling from the set of prediction errors (residuals) obtained in the past. That is, the set of prediction errors $\left\{e_{t},e_{t-1},\ldots ,e_{t-m}\right\}$ at different times is obtained from the historical observation sequence $\left\{y_{t},\;y_{t-1},\ldots ,\;y_{t-m}\right\}$ and the predicted value sequence $\left\{{\hat{y}}_{t},{\hat{\;y}}_{t-1},\;\ldots ,{\hat{\;y}}_{t-m}\right\}$ (*m* is called residual stride). A series of $e_{t+1}$ can be obtained by repeatedly sampling the prediction error set (the sampling times is denoted as B). Then, the future predicted value set $\left\{y_{t+1}\right\}$ is obtained by Equation (24).

(23) $$y_{t}={\hat{y}}_{t}+e_{t}$$

(24) $$y_{t+1}={\hat{y}}_{t+1}+e_{t+1}$$

Then, according to the confidence *k*, the quantile of $\left\{y_{t+1}\right\}$ is taken as the upper and lower limits of the prediction interval. The lower limit *L* of the interval is $quantile\left(\left\{y_{t+1}\right\},\;\left(1-k\right)/2\right)$, and the upper limit *U* is $quantile\left(\left\{y_{t+1}\right\},\;1-\left(1-k\right)/2\right)$. For example, a bootstrap-based prediction interval with a confidence level of 95% has a lower bound of $quantile\left(\left\{y_{t+1}\right\},\;2.5\%\right)$ and an upper bound of $quantile\left(\left\{y_{t+1}\right\},97.5\%\right)$.

In addition, to reduce the computational complexity of the algorithm, the method for predicting the next step is the weighted average prediction. That is, the predicted value at the next moment is equal to the weighted average of the current measured value and the predicted value at the previous moment. Its calculation is shown in (25). In this paper, α is taken as 0.5.

(25) $${\hat{y}}_{t+1}=\alpha y_{t}+\left(1-\alpha \right){\hat{y}}_{t}$$

When a fault is detected at the current moment, and the data reconstruction method cannot correctly isolate the fault, the multiple FIR algorithm is activated. That is, the above bootstrap-based confidence interval prediction method is applied to estimate the confidence interval of the predicted value at the current moment. This interval is considered as the fluctuation range of the normal measured value. When the measurement value of several sensors in the sensor array exceeds the upper or lower limit of the corresponding interval, it is considered that the current sensor is faulty, that is, the multi-fault isolation is completed. Then, the middle value (*L_i_* + *U_i_*)/2 of confidence interval corresponding to the faulty sensor *i* is used to replace the erroneous measurement value to complete the recovery of faulty data.

#### 2.3.3. Dynamic Drift Compensation

**Figure 4:**
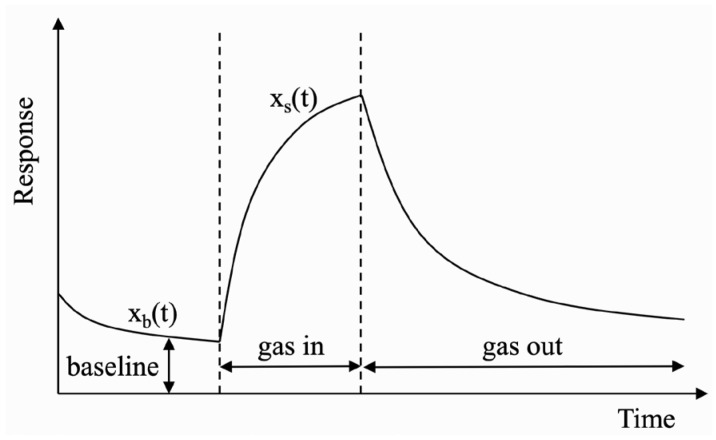
Dynamic response of gas sensor with baseline drift.

Figure 4 shows the dynamic response of a gas sensor with baseline drift. To obtain a stable sensor response, the baseline difference method shown in Equation (26) is usually used to compensate for baseline drift:

(26) $$x_{c}\left(t\right)=x_{s}\left(t\right)-x_{b}\left(0\right)$$

where $x_{s}\left(t\right)$ is the real-time response of sensor to the target gas, $x_{b}\left(0\right)$ is the estimated baseline response, and $x_{c}\left(t\right)$ is the sensor response after drift compensation. In the off-line signal preprocessing, $x_{b}\left(0\right)$ usually takes the average value of a steady-status response of sensor in pure air.

However, in the actual test environment, uncontrollable factors such as ambient temperature or humidity will cause the drift of sensor baseline, resulting in larger or smaller $x_{b}$ of different sensors in the array. At this time, if the initial $x_{b}\left(0\right)$ is still used for compensation, an error response including baseline drift will be obtained. Therefore, we propose a dynamic drift compensation method that can be operated online. It uses a dynamic baseline matrix to update the baseline response in the current environment in real time, and then performs differential processing on the current data according to the baseline matrix:

(27) $$X_{c}=X_{s}-X_{b}$$

where $X_{s}$ and $X_{c}$ are the real-time response matrix and the compensated response matrix of the sensor array, respectively, $X_{b}$ is the dynamically updated baseline matrix, the size of these matrices is 1 × *n*, *n* represents the number of gas sensors.

Baseline drift is a slowly changing process, and short-term effects can be temporarily ignored. A dynamic update step (*stride*) is added to reduce the frequency of updating the baseline matrix, thereby reducing the computational cost. In addition, when the sensor array fails, the recovered value of faulty data may be lower than the actual baseline response. To avoid the influence of the fault recovery data on the baseline matrix, a parameter (*fault_flag*) is added to control the baseline matrix $X_{b}$ to be updated when there is no failure.

**Figure 5:**
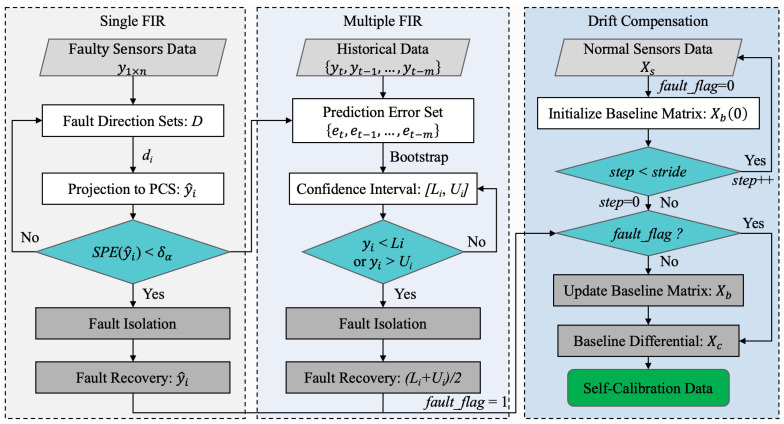
Flowchart of Self-Calibration.

In summary, the flow of the Self-Calibration algorithm is shown in Figure 5. The proposed Self-Calibration method will go through three subprocesses: single FIR, multiple FIR, and drift compensation successively from left to right. The specific step of the first two subprocesses is as follows:

(1) For faulty data, iterative data reconstruction is first performed on the set *D* of fault directions. The fault isolation is completed until the SPE statistic of the reconstructed value is lower than the threshold. Then, the reconstructed value ${\hat{y}}_{i}$ is used to recover the faulty data;
(2) If the SPE value is still greater than the threshold after reconstruction in all single-fault direction sets, then the multiple FIR based on confidence interval prediction will be carried out;
(3) Combined with the historical data within the set step size *m*, bootstrap is used to predict the confidence interval of the current measurement value. If the output of sensor is out of range, the current sensor is isolated, and the interval middle value (*L_i_ + U_i_*)/2 is used for data recovery.

Finally, the baseline drift is compensated for the fault recovery data or normal data. The specific process is as follows:

(1) Initialize the baseline matrix $X_{b}\left(0\right)$ using the response of the sensor array in pure air;
(2) Determine whether the current *step* is less than the update step *stride*. If yes, the drift compensation will not be performed at the current moment. The data will be received again, and the *step* value will be increased. If not, judge whether the current data are the fault recovery data and initialize *step* to zero;
(3) If the current *fault_flag* is 1, a fault has occurred. The update process of the baseline matrix is skipped, and the baseline difference is processed directly. If *fault_flag* is 0, it means that drift compensation is being performed on normal data, and the baseline matrix will be updated at this time.

## 3. Experimental Results and Discussion

To verify the effectiveness of the Self-Detection and Self-Calibration strategy proposed in this paper, the performance analysis and board-level verification were carried out. The output response of the sensor array used in experiments were all collected from the real mixed gas environment. Section 3.1 introduces the construction of the experimental platform and the collection of the dataset. Section 3.2 and Section 3.3, respectively, conducted comprehensive performance tests and comparisons on the data preprocessing, fault detection, fault isolation and recovery, and drift compensation algorithms applied in Self-Detection and Self-Calibration methods using different indices. Section 3.4 examines the deployed and tested complete Self-Detection and Self-Calibration methods on a resource-limited embedded hardware platform (CC1350), thereby proving the advantages of our method in speed, power consumption and resource occupation on the terminal equipment of IoT or edge computing.

### 3.1. Experimental Setup

**Figure 6:**
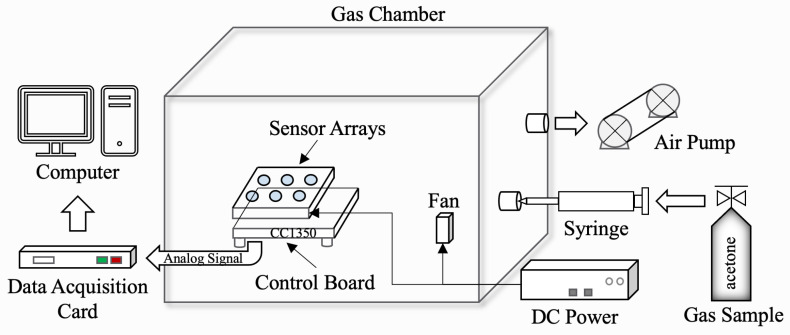
Block diagram of Self-Detection and Self-Calibration experimental platform.

**Figure 7:**
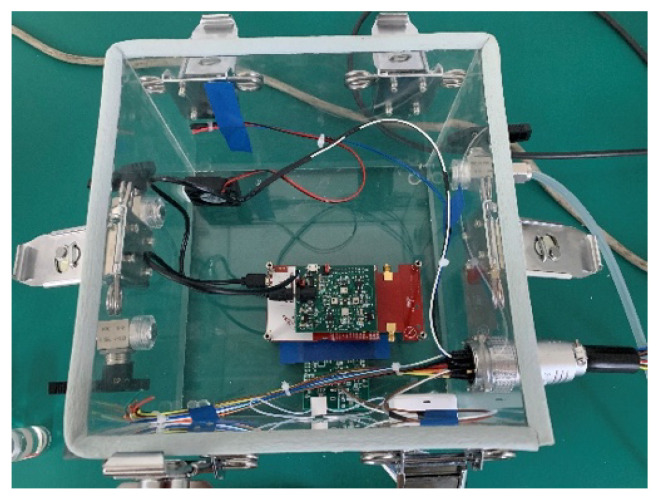
Gas chamber with sensor arrays and control board.

We built the experimental platform as shown in Figure 6. The platform was used to collect gas sensor response data and verify the capability of the proposed Self-Detection and Self-Calibration strategy. The combination of MEMS sensor array and control unit was placed in a sealable gas chamber (Figure 7). The gas chamber was equipped with a fan which was used to accelerate the diffusion of the gas. The devices in the chamber were powered by an external 5 V DC power supply. After the gas was injected into the gas chamber using a syringe, the conductivity of the sensor sensitive material would change. Then, the gas variation was converted into an analog signal through the measurement circuit for acquisition. The air pump was used to clean the gas chamber after each test.

The control board used CC1350 MCU of Texas Instruments (TI). The CPU clock frequency of CC1350 is 48 MHz and is equipped with 128 KB FLASH and 28 KB RAM. It was used for data communication with PC during data acquisition. In fact, the main function of CC1350 was to be used to simulate the control system of the actual IoT terminal devices to verify the applicability of our lightweight strategy on resource-limited platforms.

Once all equipment was connected and checked, the collection of gas response data began. The dataset collected in this paper was the sensor response data of mixed gas for diabetes exhalation diagnosis. We used four MEMS gas sensors (GM-512B, GM-802B, GM-302B, GM-502B) to form a non-redundant sensor array. Although each sensor had different sensitivity characteristics, they were all responsive to acetone. The gas composition and concentration ranges referred to the composition of the exhaled gas of diabetic patients [47]. The target gas was acetone, the concentration range was 0.2 ppm~5.0 ppm, and the response data of 5 calibration points (0.2/0.5/0.8/2.5/5.0 ppm) were collected. The specific process of data collection was as follows:

(1) Before the test, preheat the sensor array to a certain extent;
(2) Place the sensor in pure air, that is, do not inject the target gas into the gas chamber. Wait until the data are stable before collecting. The data collected at this time serves as the baseline response of the sensor array, which could be used to simulate constant output fault and initialize the baseline matrix;
(3) Inject the target gas to be measured and use a fan to evenly distribute the gas in the gas chamber. Then, the changing gas response data are collected on the PC through the data acquisition card. During the acquisition process, some samples of broken-circuit faults can be obtained by disconnecting the power supply, which could provide a reference for the generation of subsequent faulty testing samples;
(4) After collection, use the air pump to discharge the target gas in the gas chamber and open the intake valve to let the air into gas chamber. This process repeats until the response of sensors approach the baseline, thereby restoring the initial test environment.

**Figure 8:**
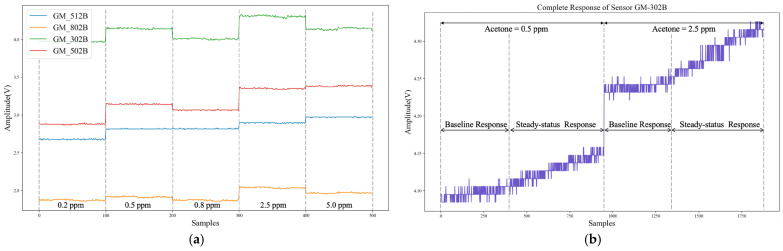
Collected Response Data of MEMS Sensor Array: (**a**) Steady-status sensor response at different acetone concentrations; (**b**) Complete Response of Sensor (GM-302B) under 0.5 ppm and 2.5 ppm.

Figure 8a shows the partial collected steady-status response data of the sensor array under normal working status. The dataset as shown in Figure 8a contained the output steady-status signals of four different MEMS gas sensors under different acetone concentrations (0.2/0.5/0.8/2.5/5.0 ppm), which would be used to train the Self-Detection model. Figure 8b shows the complete response data of the sensor GM-302B including baseline and steady-status process, which would be used to validate the proposed drift compensation algorithm. It can be clearly seen from Figure 8b that the baseline response will change at different times and different gas concentrations. In addition, the faulty data of the sensors were difficult to obtain in actual acquisition process. Therefore, 1200 faulty testing samples were randomly generated through the fault stack-based approach [19,20,29], including 300 each of impulse faults, bias faults, constant output faults and broken-circuit faults, as the testing dataset of the Self-Detection and Self-Calibration methods. Among them, the impulse and bias fault superimpose 4~5% of the average value of signal amplitude. The constant output fault was the steady response in pure air, and the broken-circuit fault was simulated by opening the electrode [19,29]. Constant output and broken-circuit faults can be regarded as special bias faults with a baseline response and close to zero magnitude. Therefore, these four different faults can also be divided into transient faults and persistent faults.

It should be noted that Section 3.2 and Section 3.3 were completed on PC. In addition to collecting and constructing training datasets, PC was also used to train Self-Detection and Self-Calibration models and for performance analysis and comparison of algorithms. CPU of PC was Intel Core i5-8257U 1.40 GHz. Section 3.4 applied the parameters of trained model to deploy and test algorithms on CC1350.

### 3.2. Self-Detection of Different Sensor Fault Types

Self-Detection methods include two parts: data preprocessing and the PCA fault detection model. Data preprocessing is the processing of singular points and random noise. Firstly, to verify the detection performance of Z-Score and Isolation Forest for outliers in dataset, 5, 10, 15, 20, 50, and 100 outliers were added to the dataset, respectively. Table 2 shows the detection rate (DR) and false positive detection rate (FPR) of these two algorithms for outliers.

If there were many singular points in the dataset, it would affect the mean and variance of data, thereby affecting the DR of Z-Score. The detection of normal data by Z-Score was rarely wrong. Isolation Forest was more suitable for continuously changing data, but the gas sensor response data fluctuated slowly, and Isolation Forest was prone to overfitting during training, resulting in false detection. Although Isolation Forest worked better when the number of outliers was large, the number of outliers in the actual dataset was generally less. Moreover, Isolation Forest had multiple hyperparameters, which were complex to adjust. Therefore, we adopted Z-Score for novelty detection of the dataset.

**Figure 9:**
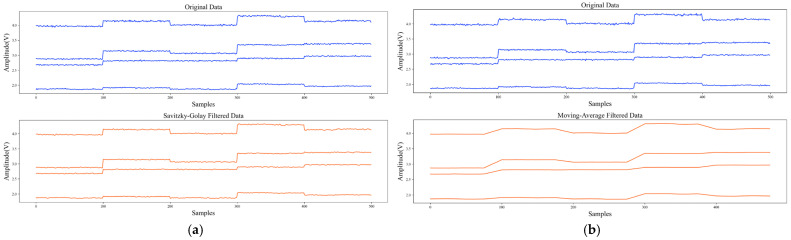
Original data and filtered data: (**a**) Signal waveforms before and after Savitzky–Golay filtering; (**b**) Signal waveforms before and after Moving-Average filtering.

To compare the filtering performance of Savitzky–Golay and Moving-Average filter, 50 dB Gaussian noise was added to the original dataset. Table 3 uses the SNR and R introduced in Section 2.2.1 to compare their filtering performance and ability to preserve the original features of data. The signal waveforms before and after Savitzky–Golay and Moving-Average filtering are shown in Figure 9. It can be found that the SNR and R of Savitzky–Golay filter were higher than those of the Moving-Average filter, which means that it could better retain the original characteristics of data while filtering out noise. It can also be seen from Figure 9 that Moving-Average filter would lead to distortion of the signal waveform. In addition, to better preserve the original features of data, we used piecewise Savitzky–Golay filtering. Mirror processing was used for border points, which could avoid boundary effect during filtering. The stride and polynomial order of Savitzky–Golay were 5 and 3, respectively, and the window length of Moving-Average was 25 in the experiment.

The steady-status outputs of the sensor array under normal operation at different calibration points were used as the training set for the PCA fault detection model, and the generated fault samples were used for the testing set. Table 4 shows the DR and FPR of different fault types using fault detection models with different statistics (SPE or T^2^). It can be found that the PCA model based on SPE (PCA-SPE) could identify four different types of faults more accurately, and the detection performance was better than the model based on T^2^. In addition, the applied data preprocessing techniques enabled the model to have no false positives during testing.

**Figure 10:**
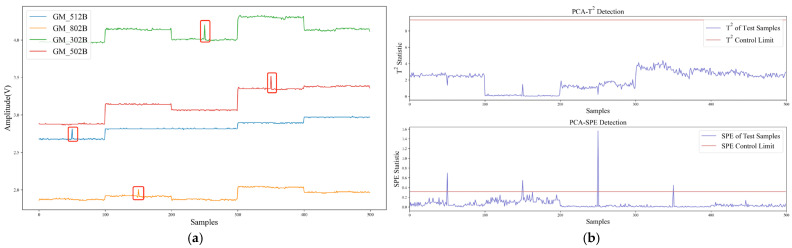
Detection results of transient faults with PCA model: (**a**) Transient Faults of Gas Sensor Array; (**b**) Detection Results of PCA-SPE and PCA-T^2^.

**Figure 11:**
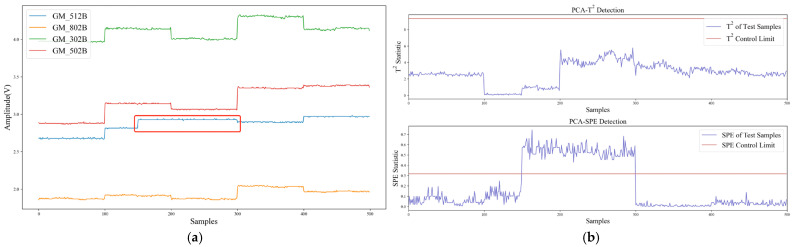
Detection results of persistent fault with PCA model: (**a**) Persistent Fault of Gas Sensor Array; (**b**) Detection Results of PCA-SPE and PCA-T^2^.

Figure 10 and Figure 11 show the detection performance of the PCA model on transient faults (impulse) and persistent faults (bias), respectively. Among them, Figure 10a marks four impulse faults (the 50th, 150th, 250th, and 350th samples), and Figure 11a marks one bias fault (the 150th~300th samples). The SPE statistics in Figure 10b and Figure 11b rose substantially and exceeded the control limit when a fault occurred. Although T^2^ statistic would also change, it was still less than the control limit in most cases, so it is difficult to detect faults.

Table 5 verifies that when there were outliers in the dataset (4 outliers were added), the performance of fault detection model would be severely degraded. This is because outliers would raise the control limit of the statistic, thereby reducing the fault detection rate.

To illustrate the impact of random noise on fault detection, Table 6 compares the change in detection rate of impulse faults when using normal data, noisy data, and filtered data. It can be seen from Table 6 that noise had a great influence on the detection of impulse faults. This is because noise increased the tolerance of model, which made some failures difficult to detect. After Savitzky–Golay (S-G) filtering, the fault detection performance returned to normal. The performance of Moving-Average (M-A) filtering was seriously degraded because it destroyed the original data features. Other types of faults had a large SPE variation, so they were not easily affected by noise, which are not mentioned in Table 6. However, when the noise increased to a certain extent, it would also be affected. This proves that data preprocessing for outliers and noise is necessary.

### 3.3. Self-Calibration of Fault Signals

Self-Calibration methods include FIR and dynamic drift compensation. Fault isolation and recovery has designed separate methods for single fault and multiple faults. The experiments in this section were also divided into tests on single-fault and multi-fault samples. The three indexes of fault isolation accuracy, Mean Absolute Error (MAE) and Mean Absolute Percentage Error (MAPE), were used to evaluate the performance of the proposed algorithms and other common FIR algorithms. The first of which represents the performance of fault isolation, and the latter two measures the accuracy of fault data recovery.

#### 3.3.1. FIR of Single Sensor Fault

Firstly, Self-Calibration applied data iterative reconstruction based on SPE statistics (SPE-IR) to implement single FIR. Table 7 compares the single-fault isolation accuracy of data iterative reconstruction methods using different statistics (SPE or T^2^) and contribution plots method. Table 8 compares the data recovery performance of different reconstruction methods. From the comparison results in Table 7 and Table 8, it can be found that the average isolation accuracy of SPE-IR was higher than that of the T^2^-based iterative reconstruction (T^2^-IR) and contribution plots method. Additionally, the data recovery accuracy of SPE-IR was also better than T^2^-IR and general reconstruction method (iterative once).

**Figure 12:**
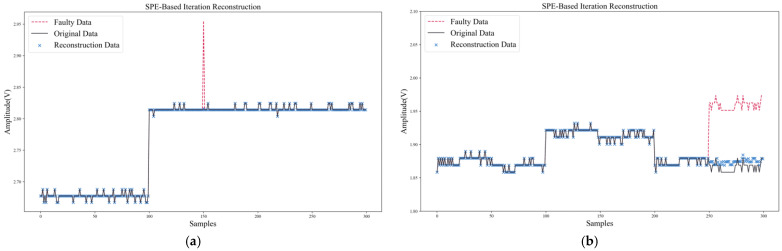
Data recovery of SPE-IR: (**a**) Transient fault; (**b**) Persistent fault.

The data recovery waveforms of SPE-IR for transient fault and persistent fault are shown in Figure 12. SPE-IR could successfully isolate and recover the error response of the faulty sensor, which proves the effectiveness of the SPE-IR method for single-fault isolation and data recovery.

In addition to comparing SPE-IR with other data reconstruction methods, two typical algorithms (ANN, RVM) for data recovery were also compared. Unlike data reconstruction, ANN and RVM used historical data to build an updating model for each sensor. When recovering the faulty data, the models needed to be updated online and make predictions.

**Figure 13:**
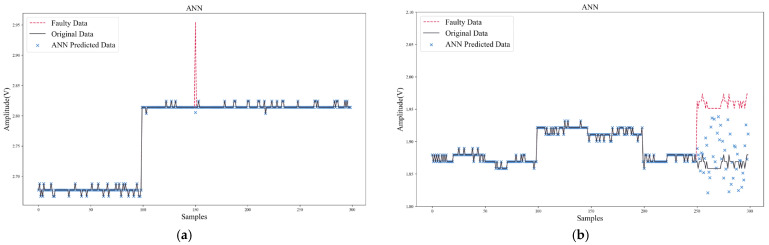
Data recovery of ANN: (**a**) Transient fault; (**b**) Persistent fault.

**Figure 14:**
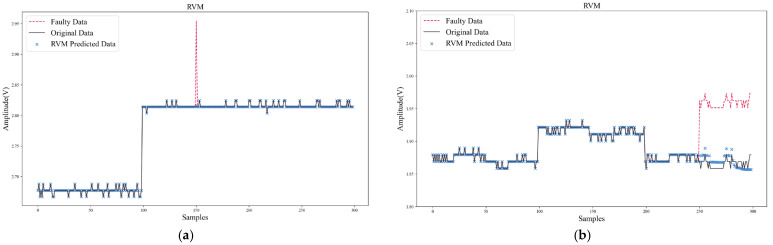
Data recovery of RVM: (**a**) Transient fault; (**b**) Persistent fault.

Figure 13 and Figure 14 show the data recovery performance of ANN and RVM. As shown in Table 9 and Table 10, to make a more comprehensive comparison of algorithm performance, in addition to comparing the data recovery error, the computation speed and model size were also compared to verify the feasibility of the algorithm being deployed on IoT terminal platforms. The hyper-parameters were set as follows: ANN’s update stride was 20, hidden layer was (100, 50), activation function was ReLu, optimizer was Adam. RVM’s update stride was 20 and kernel function was RBF. Model size in Table 10 only counted the parameter size during algorithm inference. The storage resources occupied by the storage of intermediate results and the training process were not considered, so the real storage resources occupied by ANN and RVM would be larger.

From the comparison results in Table 9 and Table 10, it can be found that SPE-IR could achieve better recovery accuracy for both transient and persistent faults. In addition, the computation speed of SPE-IR could meet the real-time requirements, and its model size was also much smaller than that of ANN and RVM. However, ANN took a long time to train and needed enough samples (200~300) to obtain better prediction performance. Moreover, ANN was prone to fall into local minimum, which made it difficult to converge effectively. Especially for persistent faults (Figure 14b), ANN could only achieve a certain degree of effective recovery within a very limited step size, after which the predicted data would diverge and deviate from the normal range.

Compared with ANN, the modeling and computation time of RVM were greatly reduced, and the data recovery accuracy was also better than that of ANN. However, the performance of RVM was still not as good as SPE-IR due to the online update of model and complex computation of kernel function.

The weak change of sensitive unit output responses in the gas sensor array made it difficult for machine learning algorithms (e.g., ANN, RVM) to extract valid features for learning from a limited number of samples. While using more samples might improve performance, it would inevitably bring greater storage and computational burdens.

#### 3.3.2. FIR of Multiple Sensor Faults

**Figure 15:**
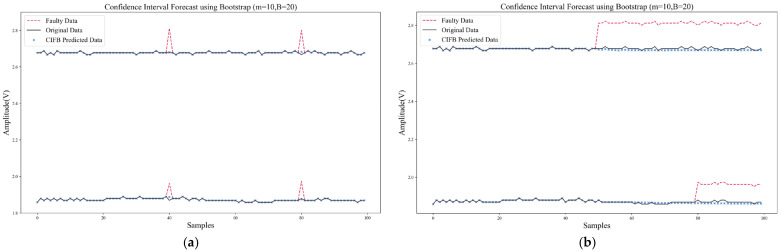
Multi-faults isolation and recovery of CIFB: (**a**) Multiple transient faults; (**b**) Multiple persistent faults.

This section used the proposed Confidence Interval Forecast using Bootstrap (CIFB) method to conduct experiments of multiple FIR. The algorithm performance, computing speed, and model size were tested and compared. In the experiment, two kinds of multi-fault samples, superimposed transient faults, and superimposed persistent faults were tested, respectively. Figure 15 shows the waveforms of multiple FIR based on CIFB. It can be seen that CIFB could effectively isolate and recover both multi-fault samples. It should be noted that when CIFB performed FIR, two parameters (residual stride *m* and sampling length *B*) needed to be selected. If *m* and *B* were too small, the prediction interval would be shifted due to incomplete sampling of the residual distribution. The larger *m* and *B*, the higher the computing time and storage usage. After tuning, the hyper-parameters of CIFB were set as follows: *m* was 10, *B* was 20, and confidence *k* was 95%.

**Figure 16:**
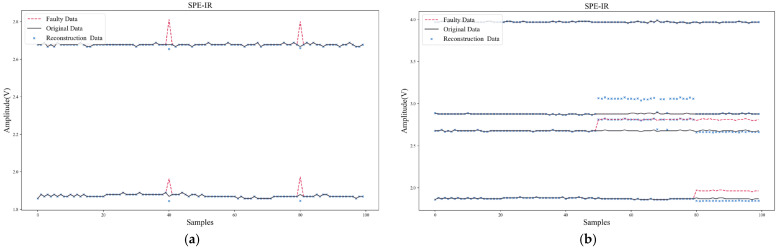
Multi-faults isolation and recovery of SPE-IR: (**a**) Multiple transient faults; (**b**) Multiple persistent faults.

Figure 16 shows the results using SPE-IR in case of multiple faults. When solving multiple FIR, SPE-IR would reconstruct on all possible fault direction sets and select the direction set with the smallest SPE statistic after reconstruction for data recovery. The shortcomings of this method are: (1) The accuracy of multi-fault isolation is low, and it is prone to fault direction judgment errors. As shown in Figure 16b, SPE-IR incorrectly judged the faulty sensor when faced with multiple persistent faults; (2) the data recovery accuracy is low. Other faulty data will be used when reconstructing sensor data in a certain direction, which can result in errors; (3) each possible fault direction set requires iteration which results in high computation latency. Therefore, in our Self-Detection and Self-Calibration strategy, the combination of SPE-IR and CIFB is used for fault isolation and recovery. In this way, single or multiple faults can be effectively solved.

Furthermore, Table 11 compares the computation time and model size of CIFB with ANN, RVM, and SPE-IR. These two metrics are the key factors that determine whether the algorithm can run efficiently on the target platform. The computation time here refers to the isolation and recovery time for multiple transient faults. The model size of ANN or RVM also needs to be multiplied by the number of sensors in the array, since it builds a separate model for each sensor. It can be found from the comparisons that CIFB had a lower recovery error, and the computation speed and model size were also much lower than those of commonly used methods (ANN, RVM).

#### 3.3.3. Drift Compensation

**Figure 17:**
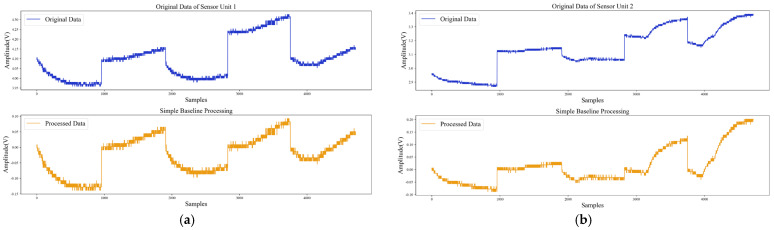
Simple baseline processing: (**a**) Drift compensation for sensor unit 1; (**b**) Drift compensation for sensor unit 2.

Although the true baseline values and responses are unknown, sensor response curves before and after drift compensation show the performance of the method used. Figure 17 shows the results of conventional baseline processing of different sensor responses using Equation (26) (the baseline values were the average response values in pure air). The raw data of sensor units 1 and 2 were response data before and after adding target gas at different concentrations. The baseline response was changing before the addition of gas, which represented the sensor had baseline drift. After simple baseline differential processing, although the response amplitude had decreased, the baseline drift still existed, and the processed data still had negative values which was unrealistic.

**Figure 18:**
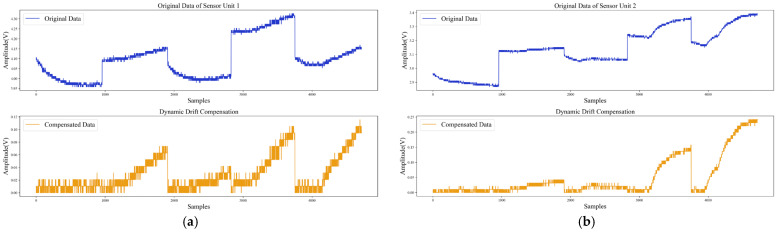
Dynamic drift compensation: (**a**) Drift compensation for sensor unit 1; (**b**) Drift compensation for sensor unit 2.

Figure 18 shows the results of processing the raw data with the proposed dynamic drift compensation method. It can be found from Figure 17 and Figure 18 that the data after dynamic compensation could better suppress the baseline drift. Moreover, the response, processed with dynamic drift compensation, was closer to the standard sensor response curve, and there were no negative values in the compensated data. The experimental results prove that the proposed method could effectively compensate for baseline drift.

### 3.4. Board-Level Verification

IoT devices such as intelligent gas sensing systems usually choose low-cost and resource limited MCUs as control chips [1,48,49]. The computation speed and resource occupation of the algorithm on MCU determine its application value. To verify the practicality of the proposed Self-Detection and Self-Calibration strategy on IoT devices, this section deployed our algorithms on CC1350 MCU and compared it with other existing algorithms (ANN, RVM).

Table 12 shows the time periods and running time (clock frequency is 48 MHz) of Self-Detection and Self-Calibration for different types of test samples (normal, single-fault, multi-fault). To obtain a more accurate measurement, the algorithm was run 10 times in a loop and the average running time was recorded. The computation time of the multi-fault testing samples in Table 12 is higher than other samples because it includes the time of updating data matrixes. Assuming that the probability of single fault and multiple faults is 5% (the real probability of faults should be much lower than this), the average running time of our method is about 0.28 ms, while the running time of ANN and RVM are 75.03 ms and 9.25 ms, respectively, which indicates that our strategy has lower computational complexity and better real-time computing performance.

Table 13 summarizes the storage resource utilization of the proposed Self-Detection and Self-Calibration strategy and other methods (ANN, RVM). The ANN and RVM methods occupied a large amount of on-chip storage resources (80.85%, 48.93%) due to complex model parameters, which would limit the deployment of other algorithms such as pattern recognition on the target platform. Meanwhile, our method only occupied very few storage resources (7.89%), which could improve the flexibility for deployment of other application algorithms.

Meanwhile, the standby power consumption of CC1350 and the running power and energy consumption of our strategy and other methods (ANN, RVM) are shown in Table 14 (Supply Voltage is 3.3 V). When testing power consumption, to measure the current of chip more accurately, we excluded the interference of peripherals and leakage current. The standby mode in Table 14 measured the current when CC1350 starts in low-power mode (RTC running, RAM and CPU retention), and the operation mode measured the average current when the algorithms were running. The running power and energy consumption of our method were 9.884 mW and 2.808 μJ. Additionally, the comparison results in Table 14 proved that our method had less energy consumption than other methods. The board-level verifications in Table 13 and Table 14 demonstrate that our method is low in resource consumption and running power, indicating that it can be efficiently deployed and run on most IoT devices.

## 4. Conclusions

To address the reliability issues of operation and measurement of MEMS gas sensor arrays, a lightweight Self-Detection and Self-Calibration strategy is proposed in this paper. Firstly, a Self-Detection model is built by employing data preprocessing and PCA techniques, which can effectively monitor the working status of the sensor array in real time. Then, when an abnormal status is detected, the Self-Calibration method combined with data reconstruction and confidence interval prediction can identify faulty sensors and accomplish failure recovery in real-time. Finally, dynamic drift compensation is used to recover the true sensor response. Experimental results indicated that our approach outperformed other typical algorithms and had better fault detection, isolation, and data recovery performance. Furthermore, since the proposed strategy can achieve Self-Detection and Self-Calibration of sensor arrays with only few computation and storage resources, it can be deployed on low-cost, low-power IoT sensing systems. It is of great significance for the proper operation of gas sensing system. In future work, we will focus on applying adaptive correction methods to enhance the performance of drift compensation.

## Figures and Tables

**Table 1:** The fault types and corresponding fault manifestations.

Fault Types	Manifestations	Causes
Impulse	Sudden change in output signal	Uncontrollable gas, power interference, etc.
Bias	Output signal deviates from the real signal	Material structure transition, supply voltage deviation, etc.
Constant	Output signal is a constant value	Gas-sensitive material pollution, shedding, etc.
Broken-Circuit	No output signal	Power failure, electrode disconnection, etc.
Drift	Output signal changes slowly	Material aging, environmental impact, etc.

**Table 2:** Comparison of different novelty detection algorithms.

Algorithms	Number of Outliers	5	10	15	20	50	100
Z-Score	DR (%)	100	100	100	100	96	96
	FPR (%)	0	0	0	0	0	0
Isolation Forest	DR (%)	100	100	100	100	100	100
	FPR (%)	25.1	14.6	8.0	2.5	1.1	0.5

**Table 3:** Comparison of different filters.

Filters	Savitzky–Golay	Moving-Average
SNR	51.7	41.4
R	0.997	0.970

**Table 4:** DR and FPR of different fault types.

Models	Fault Types	Impulse	Bias	Constant	Broken-Circuit
PCA-SPE	DR (%)	99.3	99.6	98.0	100.0
	FPR (%)	0	0	0	0
PCA-T^2^	DR (%)	12.0	33.3	33.3	100.0
	FPR (%)	0	0	0	0

**Table 5:** Influence of outliers on fault detection.

Fault Types	Impulse	Bias	Constant	Broken-Circuit
PCA-SPE (%)	0	50	50	100
PCA-T^2^ (%)	0	0	0	100

**Table 6:** Influence of random noise on fault detection.

Datasets	Normal	Noisy	S-G Filtered	M-A Filtered
DR of Impulse Faults (%)	99	95	99	33

**Table 7:** Isolation Accuracy of Different Fault Isolation Methods.

Fault Types	Impulse	Bias	Constant	Broken-Circuit	$\bar{Accu}$
SPE-IR	99	98	99	100	99
T^2^-IR	63	45	100	100	77
Contribution Plots (SPE)	58	69	58	75	65
Contribution Plots (T^2^)	16	25	38	75	38.5

**Table 8:** Data Recovery Error (MAE/MAPE) of Different Reconstruction Methods.

Figure	Impulse	Bias	Constant	Broken-Circuit	$\bar{MAE}/\bar{MAPE}$
SPE-IR	0.00176/0.0615	0.00229/0.0766	0.00481/0.1811	0.00255/0.1021	0.00285/0.1053
T^2^-IR	0.00727/0.2483	0.01611/0.5367	0.00220/0.0939	0.00255/0.1021	0.00703/0.2452
General Reconstruction	0.00624/0.2213	0.00971/0.3340	0.05600/1.9288	0.24873/8.3187	0.08017/2.7007

**Table 9:** Comparison of data recovery error (MAE/MAPE).

Methods	Transient Fault (10^−5^)	Persistent Fault (10^−3^)
SPE-IR	0.1763/6.2673	0.3839/20.6146
ANN	2.3454/83.3408	3.8944/208.7939
RVM	0.8262/29.3595	1.5227/81.5597

**Table 10:** Comparison of computation speed and model size.

Methods	Computation Speed (ms)	Model Size (Bytes)
SPE-IR	0.21	40
ANN	134.69	28.8 K
RVM	16.59	17.1 K

**Table 11:** Comparison of multi-faults computation time and model size.

Methods	CIFB	ANN	RVM	SPE-IR
Computation Time (ms)	0.65	319.75	32.35	0.68
Model Size (Bytes)	1.79 K	28.8 KB	17.1 KB	80 B

**Table 12:** Computation speed of Self-Detection and Self-Calibration on CC1350.

Testing Samples	Time Periods	Running Time		
		Ours (μs)	ANN (ms)	RVM (ms)
Normal	7010	146.04	38.56	4.75
Single-Fault	23,512	489.83	129.36	15.93
Multi-Faults	123,059	2563.73	677.17	83.41
Average	13,637	284.11	75.03	9.25

**Table 13:** Storage resource utilization of Self-Detection and Self-Calibration on CC1350.

Storage Resources	FLASH			SRAM		
	Ours	ANN	RVM	Ours	ANN	RVM
Occupied Resources (Bytes)	10.22 K	103.49 K	62.64 K	232	2.39 K	1.41 K
Total (Bytes)	128 K	128 K	128 K	28 K	28 K	28 K
Utilization Rate	7.98%	80.85%	48.93%	0.83%	8.54%	5.04%

**Table 14:** Power and energy consumption of Self-Detection and Self-Calibration on CC1350.

Mode	Standby	Operation		
		Ours	ANN	RVM
Current (mA)	0.356 μA	2.995	3.115	3.026
Power (mW)	1.173 μW	9.884	10.280	9.986
Energy (μJ)	/	2.808	771.308	92.371
